# Elevated plasma factor XI predicts cardiovascular events in patients with type 2 diabetes: a long-term observational study

**DOI:** 10.1186/s12933-023-01905-5

**Published:** 2023-07-17

**Authors:** Elżbieta Paszek, Maciej Polak, Agata Hanna Bryk-Wiązania, Małgorzata Konieczyńska, Anetta Undas

**Affiliations:** 1 Clinical Department of Interventional Cardiology, John Paul II Hospital, Krakow, 31-202 Poland; 2 Department of Thromboembolic Disorders, Institute of Cardiology, Jagiellonian University Medical College, Krakow, 31-202 Poland; 3 Department of Epidemiology and Population Studies, Jagiellonian University Medical College, Krakow, Poland; 4 Chair and Department of Endocrinology, Jagiellonian University Medical College, Krakow, Poland; 5 Department of Diagnostic Medicine, John Paul II Hospital, Krakow, 31-202 Poland; 6 Krakow Center for Medical Research and Technologies, John Paul II Hospital, Krakow, 31-202 Poland

**Keywords:** Factor XI, Type 2 Diabetes Mellitus, Myocardial Infarction, Stroke, Cardiovascular Mortality

## Abstract

**Background:**

Type 2 diabetes mellitus (T2DM) patients are at high risk of cardiovascular (CV) events. Factor XI (FXI) is associated with arterial thromboembolism, including myocardial infarction (MI), stroke, and CV mortality. The role of FXI in T2DM is unknown. We investigated whether plasma FXI is associated with CV events in T2DM patients in long-term observation.

**Methods:**

In 133 T2DM patients (aged 66 ± 8 years, 40.6% women, median T2DM duration 5 [2–10] years) we assessed plasma FXI levels, along with fibrin clot properties, thrombin generation, and fibrinolysis proteins. A composite endpoint of MI, stroke, or CV death, as well as CV mortality alone were assessed during a median follow-up of 72 months.

**Results:**

Plasma FXI above the 120% upper normal limit was detected in 25 (18.8%) patients and showed positive association with LDL cholesterol and thrombin activatable fibrinolysis inhibitor, but not glycated hemoglobin, inflammatory markers or thrombin generation. The composite endpoint (n = 21, 15.8%) and CV death alone (n = 16, 12%) were more common in patients with elevated FXI (hazard ratio [HR] 10.94, 95% confidence interval [CI] 4.46–26.87, p < 0.001 and HR 7.11, 95% CI 2.61–19.31, p < 0.001, respectively). On multivariable analysis, FXI remained an independent predictor of the composite endpoint and CV death, regardless of concomitant coronary artery disease.

**Conclusions:**

To our knowledge, this study is the first to show that in T2DM patients, elevated FXI could predict major CV events, including mortality, which suggest that anti-FXI agents might be a potential novel antithrombotic option in this disease.

**Supplementary Information:**

The online version contains supplementary material available at 10.1186/s12933-023-01905-5.

## Introduction

Cardiovascular (CV) events, including myocardial infarction (MI) and stroke, account for more than 50% of overall mortality in type 2 diabetes mellitus (T2DM) [1].

Patients with T2DM are characterized by prothrombotic alterations [2], including enhanced platelet activation, heightened thrombin generation, and the formation of denser, less porous fibrin clots, which are resistant to lysis [3], also in patients with concomitant coronary artery disease (CAD) [4]. The prothrombotic fibrin clot phenotype in T2DM is related to glycation of fibrinogen and plasminogen under chronic hyperglycemia [5, 6], plasma protein carbonylation [7], hypofibrinolysis in part driven by increased circulating plasminogen activator inhibitor (PAI-1) [8], and thrombin-activatable fibrinolysis inhibitor (TAFI) [9], as well as enhanced cross-linking of α2-antiplasmin into the fibrin network [10]. Several studies showed that the prothrombotic fibrin clot properties are associated with adverse clinical outcomes also in T2DM [11, 12]. We have recently reported that hypofibrinolysis and formation of dense fibrin clots can predict CV mortality in T2DM patients in long-term follow-up [13].

An increasing amount of data show that blood coagulation influences both the progression of atherosclerosis and its thrombotic manifestations [14]. Coagulation factor (F) XI, linking the contact system with the extrinsic coagulation pathway, has been attracting growing interest in recent years [15]. A genetic FXI deficiency was found to protect against MI, ischemic stroke, and venous thromboembolism [16]. Elevated plasma FXI, along with prekallikrein and high-molecular-weight kininogen, has been shown to increase the risk of MI [17].The RATIO study showed that plasma FXI was independently associated with an increased risk of ischemic stroke and MI in young women [18].

Activated FXI (FXIa) was found in plasma of 40% of patients with advanced CAD and correlated with unfavorable fibrin clot properties, including low permeability and prolonged lysis time [19]. We have shown recently, that in patients with advanced CAD, FXIa was an independent predictor of MI, stroke, or CV death in long-term follow-up [20]. In patients with atrial fibrillation FXIa was an independent predictor of stroke or CV mortality which occurred despite oral anticoagulation [21]. The persistent generation of FXIa in the plasma is likely driven by oxidative stress, inflammation, and endothelial injury [14], all of which are involved in the pathophysiology of DM, suggesting a possible FXI upregulation in diabetic patients. To our knowledge, there have been no published reports on the contribution of FXI levels to the risk of CV events in T2DM patients.

Given evidence for a prognostic value of high FXI in atherosclerotic vascular disease and arterial thrombosis, we hypothesized that elevated plasma FXI levels are associated with thromboembolic events in T2DM patients.

## Methods

We enrolled 156 adult patients, diagnosed with T2DM in accordance with the American Diabetes Association criteria. The study population was described previously [22]. Briefly, the exclusion criteria were as follows: acute infection, chronic inflammatory disease, recent thromboembolic events, anticoagulant treatment, known active malignancy, or advanced chronic kidney disease. We collected basic demographic and clinical data, including comorbidities, time from T2DM diagnosis, and medications used. The study protocol conformed to the ethical guidelines of the 1975 Declaration of Helsinki and was approved by the local ethics committee. Study participants provided informed written consent.

### Laboratory parameters

The laboratory measurements were described in detail before [13]. In short, fasting venous blood samples were obtained between 8:00 and 10:00 am. Basic laboratory parameters were measured using routine laboratory techniques. Fibrinogen was assayed with the von Clauss method. Plasminogen and α2-antiplasmin were measured using chromogenic assays (STA Stachrom α2-antiplasmin and plasminogen, Diagnostica Stago, Asnieres, France). Immunoenzymatic assays were used to measure tissue plasminogen activator (tPA) antigen (TintElize, Umea, Sweden), PAI-1 antigen (American Diagnostica, Stamford, CT, USA), TAFI antigen (Chromogenix, Lexington, MA, USA), and soluble thrombomodulin (Diagnostica Stago). Plasma thrombogenic potential was assessed using calibrated automated thrombography (Thrombinoscope BV, Maastricht, the Netherlands). Peak thrombin level was measured as previously described [23]. Plasma samples were analyzed in duplicates, and the intra-assay variability was 6%. Plasma fibrin clot parameters, involving clot turbidity, permeation, compaction, lysability, maximum concentration (D-D_max_), and rate of D-dimer (D-D_rate_) released during tPA-induced clot lysis, were described previously [22].

FXI was measured by a one-stage clotting assay using factor-deficient plasma (Siemens, Marburg, Germany), with a reference range of 70–120%. The assay allows for calculating FXI concentration by the measuring ability of a tested sample to correct the prolonged activated partial thromboplastin time in a standard FXI-deficient sample.

### Follow-up

We conducted follow-up during a clinic visit or by phone call. When an endpoint was recorded, the patients or their families were asked to provide medical records. Additionally, in each case of death, we performed a verification using a National Mortality Registry maintained by the State Systems Department of the Ministry of Digital Affairs, where ICD-10 codes with the cause of death are entered. The definition of CV death included death with the following International Statistical Classification of Diseases 10 (ICD-10) codes: I70.9, I25.0, I25.9, I50 and I11.0. In case of code R99, we classified the cause of death using additional clinical data. The primary composite endpoint included MI, ischemic stroke, and CV death. The secondary endpoint was CV death analyzed separately. We used The 4th Universal Definition of MI, excluding type 2 MI [24];ischemic stroke was defined based on the 2013 guidelines [25]. The expected CV mortality was 1.76 per 100 patient-years based on a recent high-volume report [26].

### Statistical analysis

Continuous variables were reported as means (standard deviation) or medians (interquartile range), as appropriate. The Kolmogorov–Smirnov test was used to test the normal distribution of variables. Categorical variables were reported as numbers and percentages. The differences in the variables were tested using the Student’s test or the analysis of variance and the χ2 test. We used the upper normal limit (120%) provided by the test’s manufacturer to discriminate between patients with normal and high FXI. Additionally, we performed a ROC analysis which confirmed this cut-off point: for the composite endpoint the highest Youden Index was 0.56 for FXI of 121.5% (sensitivity 0.62, specificity 0.94).

Predictors of FXI > 120% were assessed using multivariable logistic regression. Results of the fitted model were presented as odds ratio with 95% confidence interval (95% CI). The receiver operating characteristics (ROC) curves were used to assess the discrimination of fitted logistic models. Areas under the ROC curve (AUC) with 95% CIs were calculated. The associations between FXI and the clinical endpoints were assessed using multivariable Cox proportional hazard models. Due to a low number of events separate models for covariates were fitted. The covariates were chosen based on associations from univariable analysis and potential known confounders, based on data from previous studies. The results of Cox regression were reported as hazard ratios (HRs) with 95% CIs. The goodness-of-fit of the models was assessed using Harrell’s C-index. Two-sided p-values < 0.05 were considered statistically significant. Analyses were performed using IBM Corp. software released in 2021 (IBM SPSS Statistics for Windows; version 28.0.; IBM Corp, Armonk, NY, USA) or R Core Team 2013 (R: A language and environment for statistical computing; R Foundation for Statistical Computing, Vienna, Austria).

## Results

We enrolled 156 patients with T2DM, but 23 (14.7%) individuals were lost to follow-up. The final analysis involved 133 patients aged 66 ± 8.0 years (Table 1). At baseline the median time since T2DM diagnosis was 5 (2-10) years, and the mean glycated hemoglobin level was 6.5 (6.1–7.1) %. CAD concomitant with T2DM was observed in 64.7% of the cases. As shown in Table 1, most patients were treated with metformin (60.2%), and insulin was used in 24.8%.

**Table 1:** Baseline patient characteristics based on FXI levels

	All patients (n = 133)	FXI ≤ 120% (n = 108, 81.2%)	FXI > 120% (n = 25, 18.8%)	*P* value
Age, years, n (%)	66.0 (8.0)	65.5 (8.0)	68.5 (7.8)	0.09
Female, n (%)	54 (40.6)	40 (37.0)	14 (56.0)	0.08
Body mass index, kg/m2	31.9 (5.2)	31.8 (5.4)	32.2 (4.4)	0.78
Current smoker, n (%)	12 (9.0)	10 (9.3)	2 (8.0)	1.00
Time since diabetes diagnosis, years	5 (2–10)	5 (2–10)	5 (3–10)	0.82
**Comorbidities, n (%)**				
Hypertension	126 (94.7)	102 (94.4)	24 (96.0)	1.00
Coronary artery disease	86 (64.7)	68 (63.0)	18 (72.0)	0.39
Prior myocardial infarction	25 (18.8)	20 (18.5)	5 (20.0)	0.86
Prior stroke	2 (1.5)	2 (1.9)	0.00	
Retinopathy	21 (15.8)	15 (13.9)	6 (24.0)	0.23
Nephropathy	23 (17.3)	16 (14.8)	7 (28.0)	0.14
Neuropathy	24 (18.0)	19 (17.6)	5 (20.0)	0.78
**Pharmacotherapy, n (%)**				
Acetylsalicylic acid	105 (79.0)	85 (78.7)	20 (80.0)	0.89
Clopidogrel	10 (7.5)	9 (8.3)	1 (4.0)	0.68
Statin	106 (79.7)	85 (78.7)	21 (84.0)	0.55
Sulfonylurea	56 (42.1)	47 (43.5)	9 (36.0)	0.49
Metformin	80 (60.2)	65 (60.2)	15 (60.0)	0.97
Insulin	33 (24.8)	23 (21.3)	10 (40.0)	0.051
**Basic laboratory data**				
White blood count, x 10^9^/l	7.1 (1.4)	7.1 (1.4)	7.11 (1.6)	0.94
Neutrophiles, x 10^9^/l	60.8 (8.7)	60.5 (8.7)	62.2 (8.9)	0.38
Lymphocytes, x 10^9^/l	27.5 (7.7)	27.7 (7.7)	26.8 (8.1)	0.62
Platelets, x 10^9^/l	207 (172–254)	206 (171–255)	215 (197–254)	0.26
Hemoglobin, g/dl	13.8 (1.2)	13.8 (1.1)	13.5 (1.4)	0.18
GFR, ml/min	78.4 (20.6)	79.1 (20.1)	75.6 (23.0)	0.44
Fasting glucose, mmol/l	5.9 (5–7.4)	5.9 (5–7.35)	6.3 (5.3–7.6)	0.54
HbA1c, %	6.5 (6.1–7.1)	6.5 (6–7.25)	6.4 (6.2–7.1)	0.67
Total cholesterol, mmol/l	4.2 (3.5-5.0)	4.1 (3.5–4.8)	5.0 (4.0-5.8)	0.008
LDL-cholesterol, mmol/l	2.3 (1.9–2.9)	2.3 (1.8–2.8)	2.9 (2.1–3.6)	0.02
HDL-cholesterol, mmol/l	1.3 (1.1–1.6)	1.3 (1.1–1.6)	1.5 (1.2–1.7)	0.14
hsCRP, mg/l	1.9 (1.1–3.7)	1.8 (1.0–3.4)	3.2 (1.4–4.9)	0.06
**Coagulation parameters**				
INR	0.99 (0.95–1.03)	0.98 (0.94–1.03)	0.99 (0.96–1.01)	0.78
APTT, s	27.3 (25.9–9.1)	27.4 (26.0-29.1)	27.1 (25.7–29.1)	0.71
Fibrinogen, g/l	3.1 (0.6)	3.0 (0.6)	3.2 (0.6)	0.16
Peak thrombin, nM	235.1 (45.7)	233.5 (46.6)	242.0 (42.0)	0.40
Plasminogen activity, %	105 (98–117)	107 (98–117)	102 (97–114)	0.40
tPA antigen, ng/ml	11.5 (2.8)	11.6 (2.7)	11.3 (3.2)	0.61
PAI-1 antigen, ng/ml	32.8 (28.7–38.0)	32.6 (28.6–38.0)	33.0 (29.0–37.0)	0.86
α2-antiplasmin, %	105 (99–116)	106 (99–116)	103 (99–114)	0.79
TAFI activity, %	99 (88–109)	98 (87–106)	106 (98–111)	0.01
Thrombomodulin antigen, ng/ml	2.9 (2.4–3.5)	3.0 (2.5–3.4)	2.7 (2.4–3.6)	0.62
FXI, %	107.3 (13.0)	102.8 (9.6)	126.7 (6.6)	< 0.001
**Fibrin clot properties**				
lag phase, s	43.1 (4.5)	43.0 (4.6)	43.2 (4.3)	0.87
ΔAbs max, 405 nm	0.81 (0.78–0.85)	0.81 (0.77–0.86)	0.82 (0.80–0.84)	0.57
Ks, x10^− 9^cm^2^	7.1 (0.8)	7.2 (0.9)	7.0 (0.8)	0.50
Compaction, %	44.5 (5.9)	44.9 (6.0)	42.9 (5.2)	0.14
CLT, min	94.1 (17.5)	94.0 (18.1)	94.4 (14.6)	0.91
t50_%_, min	9.9 (9.0-10.5)	9.8 (8.9–10.5)	9.9 (9.2–10.6)	0.37
D-D_max_, mg/l	3.86 (3.62–4.12)	3.86 (3.60–4.11)	3.88 (3.71–4.22)	0.24
D-D_rate_, mg/l/min	0.070 (0.005)	0.070 (0.005)	0.069 (0.005)	0.34

Abbreviations: ΔAbs – maximum absorbance at plateau; APTT - Activated Partial Thromboplastin Time; CLT – clot lysis time; D-D_max_ – maximum D-dimer concentration; D-D_rate_ – maximum rate of increased in D-dimer concentration; GFR – glomerular filtration rate (GFR = 175 × serum creatinine^− 1.154^ × age^− 0.203^ × 1.212 [if patient is black] × 0.742 [if patient is female]); HbA1C – glycated hemoglobin; HDL – high-density lipoprotein; hsCRP – high sensitivity C-reactive protein; INR – international normalized ratio; Ks – permeability coefficient; LDL– low-density lipoprotein; PAI-1 – Plasminogen activator inhibitor – 1; t_50%_ - time required for a 50% decrease in clot turbidity; TAFI – throbin-activatable fibrinolysis inhibitor; tPA – tissue plasminogen activator;

Plasma FXI above 120% was detected in 25 (18.8%) patients. When comparing patients with normal and elevated FXI, there were no differences in the clinical characteristics, basic laboratory and coagulation parameters with the exception of total and LDL cholesterol, as well as TAFI activity, which were higher in patients with elevated FXI. There was a positive correlation between FXI and total (R = 0.40, P < 0.001), and LDL-cholesterol (R = 0.35, p < 0.001). On multivariable analysis LDL-C and TAFI were associated with elevated FXI, independently of age, sex, baseline concomitant CAD, and CRP (Table 2). We did not observe correlations between FXI and any of the coagulation or fibrin clot parameters.

**Table 2:** Predictors of elevated FXI on multivariable analysis

Variable	OR	95% CI	*p* value
Age	1.04	(0.98–1.11)	0.23
Female	1.57	(0.58–4.23)	0.37
CAD	2.90	(0.92–9.17)	0.07
LDL-C	1.70	(1.01–2.86)	0.046
TAFI (10%)*	1.54	(1.10–2.16)	0.01
hsCRP	1.05	(0.93–1.19)	0.40
AUC 95% CI	0.78 (0.69–0.87)		

Abbreviations: AUC – area under the curve; CAD – coronary artery disease; LDL-C – low density lipoprotein cholesterol; hsCRP – high-sensitive C-reactive protein; OR – odds ratio; TAFI – thrombin activatable fibrinolysis inhibitor; *OR value for a 10% rise in TAFI activity

### Composite endpoint during follow-up

The composite endpoint occurred in 21 (15.8%) patients (2.86 per 100 patient-years), including one nonfatal MI, four nonfatal strokes and 16 CV deaths during the follow-up of 72 (68–74) months. As expected, patients who experienced the composite endpoint were on average four years older than the remainder (Table 3). No other differences in demographic or clinical data were noted between patients with and without the composite endpoint (Table 3). Except for a larger proportion of subjects treated with sulfonylurea and lower number of those on metformin in the former group, there were no differences in medications between the two groups (Table 3). Regarding laboratory variables, the group who experienced the composite endpoint had higher baseline white blood count and lower fasting glucose (Table 3), while the coagulation parameters, including fibrinogen, peak thrombin, plasminogen, α2-antiplasmin or TAFI activity, tPA, PAI-1, or thrombomodulin were not linked with this endpoint (Table 3). Fibrin clot properties where similar between the patients with and without the composite endpoint with the exception of higher t_50%_, higher D-D_max_, and lower D-D_rate_ in the former group (Table 3).

**Table 3:** Patient characteristics with regard to clinical endpoints

			MI + stroke + Cardiovascular death				Cardiovascular death		
	All (n = 133)		No (n = 112, 84.2%)	Yes (n = 21, 15.8%)	*p* value		No (n = 117, 88%)	Yes (n = 16, 12%)	*p* value
Age, years, n (%)	66.0 (8.0)		65.4 (7.8)	69.4 (8.4)	0.03		65.6 (7.7)	69.2 (9.4)	0.09
Female, n (%)	54 (40.6%)		47 (42.0)	7 (33.3)	0.46		49 (41.9)	5 (31.3)	0.47
Body mass index, kg/m2	31.9 (5.2)		32.2 (5.2)	30.1 (4.7)	0.09		32.1 (5.2)	30.3 (5.1)	0.18
Current smoker, n (%)	12 (9.0%)		10 (8.9)	2 (9.5%)	1.00		10 (8.5)	2 (12.5)	0.64
Time since diabetes diagnosis, years	5 (2–10)		5 (2–10)	5 (3–11)	0.86		5 (2–10)	8 (2–13)	0.38
**Comorbidities, n (%)**									
Hypertension,	126 (94.7)		108 (96.4)	18 (85.7)	0.08		112 (95.7)	14 (87.5)	0.60
Coronary artery disease	86 (64.7)		71 (63.4)	15 (71.4)	0.48		73 (62.4)	13 (81.3)	0.32
Prior myocardial infarction	25 (18.8)		19 (17.0)	9 (28.6)	0.23		19 (16.2)	6 (37.5)	0.08
Prior stroke	2 (1.5)		1 (0.9)	1 (4.8)	-		1 (0.9)	1 (6.3)	-
Retinopathy	21 (15.8)		16 (14.3)	5 (23.8)	0.33		17 (14.5)	4 (25.0)	0.28
Nephropathy	23 (17.3)		16 (14.3)	7 (33.3)	0.054		17 (14.5)	6 (37.5)	0.07
Neuropathy	24 (18.0)		20 (17.9)	4 (19.0)	1		20 (17.1)	4 (25.0)	0.49
**Pharmacotherapy, n (%)**									
Acetylsalicylic acid	105 (79.0)		90 (80.4)	15 (71.4)	0.39		92 (78.6)	13 (81.3)	1
Clopidogrel,	10 (7.5)		9 (8.0)	1 (4.8)	1		9 (7.7)	1 (6.3)	1
Statin	106 (79.7)		88 (78.6)	18 (85.7)	0.57		93 (79.5)	13 (81.3)	1
Sulfonylurea	56 (42.1)		43 (38.4)	13 (61.9)	0.045		47 (40.2)	9 (56.3)	0.22
Metformin	80 (60.2)		73 (65.2)	7 (33.3)	0.006		75 (64.1)	5 (31.3)	0.01
Insulin	33 (24.8)		27 (24.1)	6 (28.6)	0.66		27 (23.1)	6 (37.5)	0.23
**Basic laboratory data**									
White blood count, x 10^9^/l	7.1 (1.4)		7.0 (1.3)	7.8 (1.9)	0.02		7.0 (1.3)	8.0 (2.1)	0.008
Neutrophiles, x 10^9^/l	60.8 (8.7)		60.0 (8.2)	65.4 (10.1)	0.008		60.1 (8.0)	66.1 (11.1)	0.009
Lymphocytes, x 10^9^/l	27.5 (7.7)		28.3 (7.4)	23.4 (8.6)	0.007		28.3 (7.3)	22.2 (9.1)	0.003
Platelets, x 10^9^/l	207 (172–254)		208 (176–254)	203 (155–254)	0.53		208 (176–255)	186 (151–248)	0.24
Hemoglobin, g/dl	13.8 (1.2)		13.8 (1.1)	13.7 (1.5)	0.82		13.8 (1.1)	13.7 (1.4)	0.66
GFR, ml/min	78.4 (20.6)		79.4 (19.7)	73.5 (25.1)	0.24		79.3 (19.4)	72.0 (28.0)	0.18
Fasting glucose, mmol/l	5.9 (5.0-7.4)		6.0 (5.3–7.4)	5.2 (4.2–6.3)	0.04		6.0 (5.3–7.4)	5.0 (4.2–6.5)	0.06
HbA1c, %	6.5 (6.1–7.1)		6.5 (6.1–7.2)	6.6 (6.1–7.1)	0.72		6.4 (6.1–7.1)	6.7 (6.2–8.5)	0.25
Total cholesterol, mmol/l	4.2 (3.5-5.0)		4.3 (3.6–5.1)	3.6 (3.4-5.0)	0.13		4.3 (3.6-5.0)	3.4 (3.3–4.4)	0.02
LDL-C, mmol/l	2.3 (1.9-3.0)		2.4 (1.9–3.1)	1.9 (1.7–2.8)	0.08		2.4 (1.9–3.1)	1.7 (1.4–2.4)	0.007
HDL-C, mmol/l	1.3 (1.1–1.6)		1.4 (1.1–1.6)	1.2 (1.0–1.5)	0.19		1.3 (1.1–1.6)	1.2 (1.0-1.5)	0.32
hsCRP, mg/l	1.9 (1.1–3.7)		1.9 (1.0-3.4)	3.2 (1.7-5.0)	0.06		1.9 (1.0-3.4)	3.3 (1.9-6.0)	0.04
**Coagulation parameters**									
INR	0.99 (0.95–1.03)		0.98 (0.94–1.02)	1 (0.96–1.05)	0.42		0.98 (0.94–1.02)	1.00 (0.96–1.06)	0.47
APTT, s	27.3 (25.9–9.1)		27.6 (26.1–29.3)	26.4 (25.5–27.8)	0.10		27.5 (26.1–29.2)	26.5 (25.2–27.7)	0.14
Fibrinogen, g/l	3.1 (0.6)		3.1 (0.6)	3.2 (0.6)	0.15		3.1 (0.6)	3.2 (0.5)	0.35
Peak thrombin, nM	235.1 (45.7)		232.3 (44.5)	249.9 (50.5)	0.11		233.2 (45.4)	248.8 (46.8)	0.20
Plasminogen activity, %	105 (98–117)		107 (99–117)	104 (97–113)	0.42		105 (98–117)	106 (89–116)	0.48
tPA antigen, ng/ml	11.5 (2.8)		11.5 (2.6)	11.7 (3.8)	0.73		11.4 (2.6)	12.3 (3.9)	0.27
PAI-1 antigen, ng/ml	32.8 (28.7–38.0)		32.7 (28.1–38.0)	33.6 (30.5–37.0)	0.50		33 (28.6–38.0)	32.1 (29.5–37.1)	0.88
α2-antiplasmin, %	105 (99–116)		105 (98.5-114.5)	113 (100–119)	0.29		104 (99–114)	115 (101–121)	0.16
TAFI activity, %	99 (88–109)		99 (88–108)	98 (90–110)	0.91		99 (88–109)	98 (86–106)	0.72
Thrombomodulin antigen, ng/ml	2.9 (2.4–3.5)		3.0 (2.5–3.5)	2.8 (2.3–3.4)	0.42		3.0 (2.5–3.5)	3.1 (2.3–3.6)	0.91
FXI, %	107.3 (13.0)		104.9 (10.7)	120.1 (16.8)	< 0.001		106.1 (11.9)	116.5 (17.4)	0.002
FXI > 120%, n (%)	25 (18.8)		12 (10.7)	13 (61.9)	< 0.001		18 (15.3)	7 (46.7)	0.003
**Fibrin clot properties**									
lag phase, s	43.1 (4.5)		43.3 (4.5)	41.9 (4.2)	0.22		43.2 (4.6)	42.4 (3.8)	0.55
ΔAbs max, 405 nm	0.81 (0.78–0.85)		0.81 (0.77–0.86)	0.83 (0.8–0.85)	0.20		0.81 (0.77–0.85)	0.83 (0.80–0.86)	0.26
Ks, x10^− 9^cm^2^	7.1 (0.8)		7.2 (0.8)	6.8 (0.9)	0.07		7.2 (0.8)	6.8 (0.8)	0.07
Compaction, %	44.5 (5.9)		44.8 (6.0)	42.7 (5.3)	0.12		44.7(6.0)	43.4 (5.3)	0.42
CLT, min	94.1 (17.5)		94.1 (17.0)	94.2 (20.3)	0.98		94.3 (17.1)	92.6 (20.9)	0.71
t_50%_, min	9.9 (9.0-10.5)		9.8 (8.9–10.4)	10.1 (9.8–10.9)	0.03		9.8 (9.0-10.4)	10.2 (9.6–11.0)	0.06
D-D_max_, mg/l	3.86 (3.62–4.12)		3.80 (3.60–4.07)	4.09 (3.80–4.31)	0.006		3.9 (3.6–4.1)	4.2 (3.8–4.5)	0.01
D-D_rate_, mg/l/min	0.070 (0.005)		0.071 (0.005)	0.068 (0.005)	0.04		0.071 (0.005)	0.068 (0.005)	0.01

Abbreviations: see Table 1

**Fig. 1:**
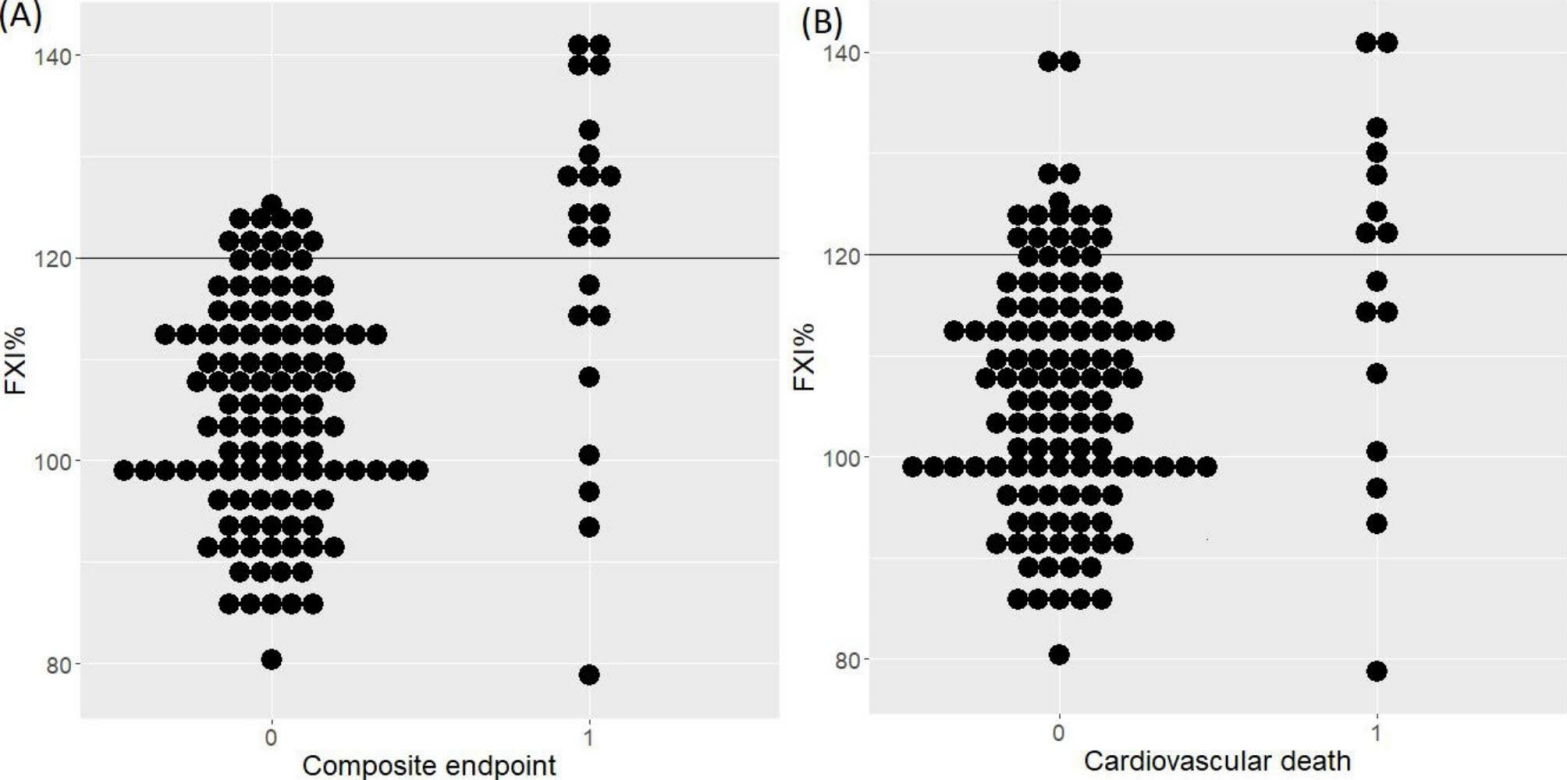
Box plots showing the distribution of plasma FXI with respect to endpoint occurrence. Calculated using the Mantel-Cox test. Abbreviations: CV – cardiovascular; MI - myocardial infarction. **A** – the composite endpoint: myocardial infarction, stroke or cardiovascular mortality, **B** – cardiovascular mortality

**Fig. 2:**
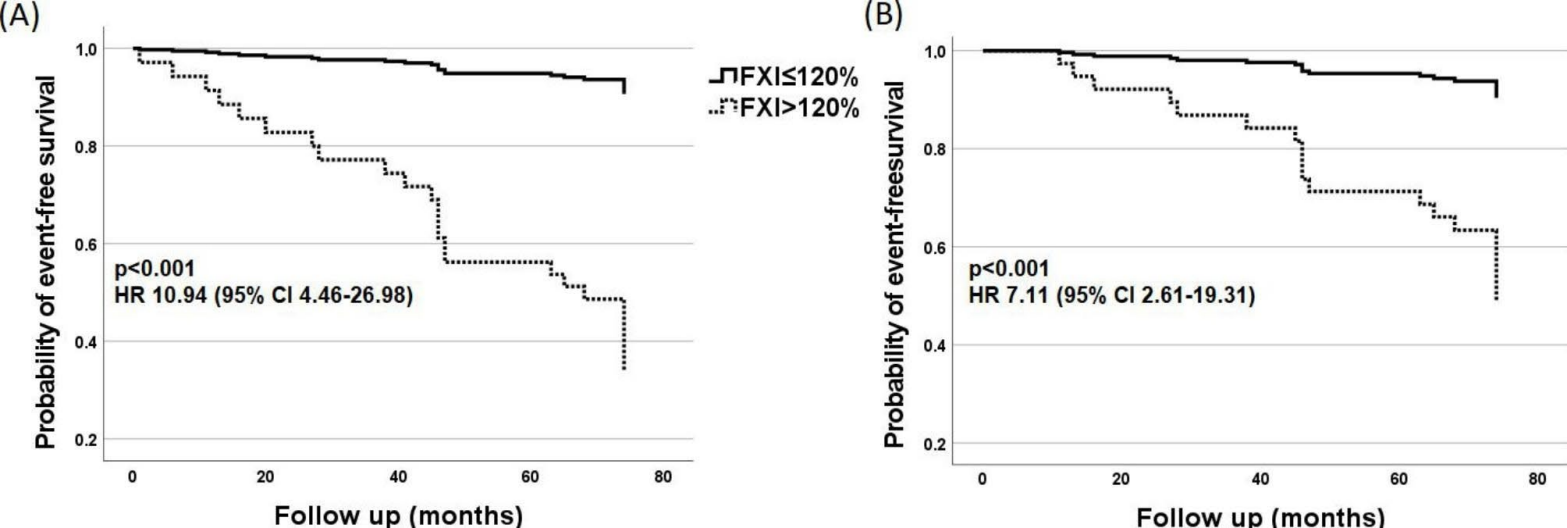
Kaplan–Meier curves showing the probability of event-free survival in stable coronary artery disease patients with regard to FXI. Abbreviations: see Figure 1

The mean FXI levels were higher in subjects with the composite endpoint (120.1 [16.8] vs. 104.9 [10.7]%, p < 0.001; Table 3; Fig. 1A), as compared with the remaining patients. Also when dichotomized, elevated baseline FXI was six times more frequent in the group with the composite endpoint as compared to the remainder (13 [61.9%] vs. 12 [10.7%], p < 0.001) and it increased the risk of its occurrence on univariable analysis (HR 10.94, 95% CI [4.46–26.87], p < 0.001, C-index = 0.76 [SE = 0.049]; Fig. 2A). Additional adjustment for age, sex, LDL-C and D-D_max_ did not attenuate the association between FXI and the composite endpoint (HR = 12.35, 95% CI (4.74–32.23), p < 0.001; C-index = 0.87 [SE = 0.044]; Table 4).

**Table 4:** Multivariable analysis – clinical endpoints

	Myocardial infarction + stroke + CV death				CV death			
	HR	95% CI	*p* value			HR	95% CI	*p* value
Age	1.08	(1.01–1.15)	0.02		Age	1.08	(0.999–1.16)	0.04
Female	0.40	(0.14–1.20)	0.10		Female	0.44	(0.12–1.52)	0.19
LDL-C	0.67	(0.38–1.16)	0.15		LDL-C	0.52	(0.25–1.04)	0.07
D-D_max_	4.97	(1.61–15.36)	0.005		D-D_max_	7.74	(2.08–28.83)	0.002
FXI > 120%	12.35	(4.73–32.23)	< 0.001		FXI > 120%	8.11	(2.82–23.20)	< 0.001
	Concordance 0.872 (0.044)					Concordance 0.851 (0.061)		

Abbreviations: see Table 1

### Cardiovascular mortality during follow-up

We recorded 16 (12%, 2.18 per 100 patient-years) cases of CV death. The patients who died of CV causes did not differ from the survivors with regard to clinical characteristics or pharmacotherapy (Table 3). Solely metformin was used less often in the group with CV death (Table 3). In comparison with the remainder, patients who suffered CV death had higher white blood cell count, higher CRP, and lower total cholesterol and LDL-C (Table 3). The patients who died from CV causes and the survivors had similar coagulation parameters and fibrin clot properties with the exception of D-D_max_ which was higher, and D-D_rate_ which was lower in the former group (Table 3).

Compared with the remainder, patients who died of CV causes had higher mean FXI (116.5 [17.4] vs. 106.1 [11.9]%, p = 0.002; Table 3; Fig. 1B) and FXI was elevated above the upper normal limit in 46.7 vs. 15.3% of the cases, p = 0.003 (Table 3). On univariable analysis FXI increased the risk of CV death (HR = 7.11, 95 CI% [2.61–19.31], p < 0.001; C-index = 0.71 [SE = 0.061]; Fig. 2B). Further adjustment for age, sex, LDL-C, and D-D_max_ did not change this association (HR = 8.11, 95% CI [2.82–23.30], p < 0.001; C-index = 0.85 [SE = 0.061]; Table 4).

### FXI in patients with concomitant CAD

Patients with concomitant CAD (n = 86), aged 67.3 ± 8.1 years, with elevated and normal FXI (n = 18, 20.9% and n = 68, 79.1%, respectively) did not differ in terms of demographic and clinical characteristics with the exception of insulin use, which was more frequent in the former group (Table S1). Patients with CAD and elevated FXI had higher CRP, higher TAFI activity and lower D-D_rate_, as compared with the remainder (Table S1).

There were 15 patients who experienced the composite endpoint in the subgroup with CAD (17.4%, 3.12 per 100 patient-years), mostly driven by CV death (n = 13, 15.1%). The patients with and without the composite endpoint were largely similar in terms of clinical characteristics, pharmacotherapy, and baseline laboratory findings (Table S2). Additionally, T2DM patients with CAD, who had an MI, stroke, or died of CV causes were more likely to have nephropathy, reflected by lower GFR (Table S2). Analysis of fibrin clot properties showed that patients with the composite endpoint had lower Ks, t50% and D-D_rate_ with a higher D-D_max_ (Table S2). Furthermore, the CAD subjects with the composite endpoint had higher baseline FXI (118.6 [13.8] vs. 105.5 [10.8] %, p < 0.001), corresponding with 60% of the patients with FXI > 120%, as opposed to 12.7% of those who did not (p < 0.001, Table S2).

The characteristics of patients with T2DM and concomitant CAD who died of CV causes vs. survivors was identical to that of the composite endpoint (Table S2 and S3). Similarly, the predictors of CV-death were the same as for the composite endpoint (Table S2 and S3).

### FXI in the subgroup without CAD

There were 47 patients, aged 63.7 ± 7.4 years, without concomitant CAD at baseline. Patients with normal and elevated FXI were similar, except for total and LDL-C, which were higher in the group with FXI > 120% (Table S4). We recorded six (12.8%) cases of the composite endpoint. There were no baseline differences between the patients with and without the composite endpoint, with the sole exception of elevated FXI, with was more frequent in the former group (66.7 vs. 7.3%, p = 0.001, Table S4).

## Discussion

To our knowledge, the current study is the first to show that in patients with T2DM elevated FXI is independently associated with the occurrence of MI, stroke, or CV death during long-term follow-up. Of note, such an association was observed also for CV mortality. A predictive value of FXI was observed in T2DM patients with and without concomitant CAD. Our findings indicate that elevated FXI could be implicated in CV events in T2DM, representing a potential therapeutic target given accumulating evidence for benefits from FXIa inhibitors in CV disease [27].

High plasma levels of fibrinogen, FVII, tissue factor, and FXII, accompanied by higher activity of tissue factor-FVIIa complex activity, are well-documented in DM [28]. Patrassi et al. studied 26 DM patients and showed elevated FXI, along with prekallikrein, and FXII [29]. In our cohort almost one in five patients had baseline FXI > 120%. The mechanisms behind this observation are obscure and probably multiple. Plasma FXI is synthesized primarily by hepatocytes, where its expression is regulated by hepatocyte nuclear factor 4α (HNF-4α), a transcription factor common to a number of other coagulation factors (VII, IX, and X) and apolipoproteins (AI, AII, AIV, B, and CIII) [30]. As shown in a previous study, inflammation, endothelial injury, and oxidative stress drive the activation of FXI in the plasma [31]. We observed a trend for higher CRP in patients with elevated FXI, which achieved the level of statistical significance in the subgroup with CAD, suggesting that chronic, low grade inflammation present in both T2DM and CAD may contribute to elevated plasma FXI. The pro-thrombotic state represented by elevated FXI may also result from endothelial dysfunction under chronic hyperglycemic conditions. Another potential factor affecting FXI levels could be glycemia control. The NEO study, performed in over 5700 middle-aged participants, including 3% diabetic patients, indicated that FXI levels rose with increasing fasting and postprandial glucose, as well as HbA1C, however the observed effects were attenuated after adjustment for total body fat [32]. We did not observe any correlation between fasting glucose or HbA1C and FXI levels in T2DM patients.

We have shown a weak, positive correlation between total cholesterol as well as LDL-C, and FXI in T2DM patients, which is consistent with our previous report on CAD patients, including 25% with concomitant DM [33]. Genes encoding FXI, other coagulation factors, and a number of apolipoproteins share the transcription factor HNF-4α. Of note, the observed association between elevated FXI and high risk of CV events was independent of LDL-C, suggesting a mechanism that is unrelated to lipid control.

We observed higher baseline TAFI activity in patients with elevated FXI, which is consistent with previous studies [34] and that plasma from FXI-deficient patients exhibited enhanced fibrinolysis due to decreased TAFI generation, as well as resistance to TAFI [35].

Given the role of FXI in the formation of thrombin following activation by FXII upon contact with negatively charged molecules, such as extracellular vesicles or misfolded proteins [36], the association of high plasma FXI levels with thromboembolic events in T2DM patients is to be expected. In vitro studies show that the more thrombin is available when clotting is initiated, the denser and lysis-resistant fibrin clots are formed [37]. In fact, we have recently shown an association between detectable FXIa and a prothrombotic fibrin clot phenotype, including increased density and stability [19]. In light of the above, it is tempting to propose that blocking FXI could be a way to prevent CV events in high-risk T2DM patients. Anti-FXI agents were proven to be safe and effective in the prevention of thromboembolism in a number of clinical settings [27], however, currently they have not been studied in T2DM.

The associations between high leukocyte count, CRP, low LDL, as well as the concentration and rate of D-dimer release during tPA-induced clot lysis (D-D_max_, D-D_rate_), and CV events, were previously reported and discussed [13]. In both lysis assessment methods, which are based on D-dimer release, and in turbidity analysis (t_50%_), thrombin is used as a coagulation trigger. Thrombin is known to activate platelet-bound FXI, and promote FIX activation, resulting in a further, explosive generation of thrombin [38]. Therefore, we hypothesize that FXI may affect thrombin-triggered coagulation and lysis. We did not observe a correlation between FXI and any of the lysis parameters, which is probably due to the fact that thrombin used in the in vitro assays would override any influence that FXI may have on these parameters. Regarding therapy, we confirmed that the use of sulfonyloureas, as well as underuse of metformin, may predispose to poor outcomes, which is consistent with previous studies [39]. In line, individuals with the composite endpoint had lower fasting glucose as compared to the remainder, which also has been already observed [40].

A relatively small difference (ca. 20%) in mean FXI values between the groups with FXI below and above 120% had a large impact on clinical outcomes, suggesting that the role of FXI may span beyond strictly coagulation. In fact, a recent proteomic study showed that in patients with venous thromboembolism (both in the acute phase and in long term follow-up) FXI was associated with a number of proteins involved in inflammation, apoptosis/tumor necrosis factor-α signaling, LDL-C receptors and oxidation, protein misfolding, and extracellular matrix interactions [41]. These are in line with the mechanisms of FXI activation and suggest that FXI may play a multifaceted role in a number of pathological pathways, making it an attractive target for further research.

### Study limitations

This study cohort was small, but our study group was representative of a T2DM population recruited six years ago. At baseline, we did not screen asymptomatic patients for CAD, hence it is possible that some cases remained undiagnosed. Due to a low number of patients with endpoints, we were able to build multivariable models which incorporated only a limited number of potential confounders. However, regardless of what potentially confounding factors were included in the models, the strength and direction of the FXI association with the endpoints remained the same.

In terms of T2DM therapy, the currently recommended sodium-glucose cotransporter inhibitors, or glucagon-like peptide-1 receptor antagonists, which may influence the risk of long term adverse events [42], were not used in the study group at the time of patient recruitment. Also, we did not assess patient compliance with the prescribed medications and glycemic control during follow-up. Nevertheless, the median T2DM duration at baseline was 5 years, hence consequences of any possible periods of inadequate glycemic control during follow-up would likely be outweighed by the effect of disease duration.

## Conclusion

This is the first study to show that in long-term follow-up elevated FXI is a strong, independent predictor of arterial thromboembolic events, including CV death, in patients with T2DM, regardless of concomitant CAD. Several trials showed an additional benefit from adding anticoagulants to aspirin in reducing the risk of MI, stroke, or CV mortality in patients with DM and CV disease [43]. In the light of our findings, we speculate that novel anti-FXI agents [27] may constitute an attractive addition to standard pharmacotherapy in T2DM patients, who are at high risk of arterial thromboembolism.

## Electronic supplementary material

Below is the link to the electronic supplementary material.

Supplementary Material 1

## Data Availability

The datasets are available from the corresponding author on reasonable request.
