# Supplementary material for: Elevated plasma factor XI predicts cardiovascular events in patients with type 2 diabetes: a long-term observational study
Source: Cardiovasc Diabetol. 2023 Jul 17;22:182. doi: 10.1186/s12933-023-01905-5 (PMC10353137; doi:10.1186/s12933-023-01905-5)
Supplement: Supplementary file 1 — Supplementary Material 1 [file 12933_2023_1905_MOESM1_ESM.docx]

**Supplementary material**

**Table S1 Baseline patient characteristics with regard to FXI levels in patients with type 2 diabetes mellitus and coronary artery disease**

|  | All patients with CAD  (n=86) | | FXI ≤ 120%,  n=68 (79.1%) | | FXI >120%,  n=18 (20.9%) | *p* value |  |
| --- | --- | --- | --- | --- | --- | --- | --- |
| Age, years, n (%) | 67.3 (8.1) | | 66.9 (8.1) | | 68.8 (8.2) | 0.37 |  |
| Female, n (%) | 31 (36.0) | | 22 (32.4) | | 0.17 | 0.17 |  |
| Body mass index, kg/m2 | 31.2 (5.0) | | 30.8 (5.0) | | 32.9 (4.5) | 0.10 |  |
| Current smoker, n (%) | 7 (8.1) | | 5 (7.4) | | 2 (11.1) | 0.63 |  |
| Time since diabetes diagnosis, years | 6 (3 - 10) | | 5 (3 - 10) | | 9 (4 - 11) | 0.19 |  |
| **Comorbidities, n (%)** |  |  |  |  |  |  |  |
| Hypertension | 79 (91.9) | | 62 (91.2) | | 17 (94.4) | 1.00 |  |
| Prior myocardial infarction | 25 (29.1) | | 20 (29.4) | | 5 (27.8) | 0.89 |  |
| Prior stroke | 2 (2.3) | | 2 (2.9) | | 0 (0) | - |  |
| Retinopathy | 18 (20.9) | | 12 (17.6) | | 6 (33.3) | 0.19 |  |
| Nephropathy | 16 (18.6) | | 10 (14.7) | | 6 (33.3) | 0.09 |  |
| Neuropathy | 19 (22.1) | | 14 (20.6) | | 5 (27.8) | 0.53 |  |
| **Medications, n (%)** |  |  |  |  |  |  |  |
| Acetylsalicylic acid | 79 (91.9) | | 61 (89.7) | | 18 (100.0) | 0.34 |  |
| Clopidogrel | 10 (11.6) | | 9 (13.2) | | 1 (5.6) | 0.68 |  |
| Statin | 76 (88.4) | | 61 (89.7) | | 15 (83.3) | 0.43 |  |
| Sulfonylurea | 40 (46.5) | | 34 (50.0) | | 6 (33.3) | 0.21 |  |
| Metformin | 50 (58.1) | | 40 (58.8) | | 10 (55.6) | 0.80 |  |
| Insulin | 22 (30.8) | | 13 (19.1) | | 9 (50.0) | 0.01 |  |
| **Basic laboratory data** |  |  |  |  |  |  |  |
| White blood count, x 10^9^/l | 7.3 (1.5) | | 7.3 (1.4) | | 7.3 (1.7) | 0.93 |  |
| Neutrophiles, x 10^9^/l | 61.3 (9.4) | | 61.2 (9.2) | | 61.9 (10.1) | 0.79 |  |
| Lymphocytes, x 10^9^/l | 26.9 (8.2) | | 26.9 (7.9) | | 26.9 (9.4) | 1.00 |  |
| Platelets, x 10^9^/l | 206 (176 - 252) | | 206 (174.5 - 257) | | 206 (178 - 249) | 0.90 |  |
| Hemoglobin, g/dl | 13.8 (1.2) | | 13.9 (1.1) | | 13.5 (1.4) | 0.27 |  |
| GFR, ml/min | 77.3 (21.3) | | 78.7 (20.4) | | 71.9 (24.1) | 0.23 |  |
| Fasting glucose, mmol/l | 5.7 (4.9-7.2) | | 5.5 (4.9-7.1) | | 5.8 (4.9-7.7) | 0.40 |  |
| HbA1c, % | 6.6 (6.1-7.3) | | 6.6 (6.1-7.3) | | 6.6 (6.3-7.4) | 0.42 |  |
| Total cholesterol, mmol/l | 4.0 (3.4-4.8) | | 3.9 (3.4-4.6) | | 4.6 (3.5-5.3) | 0.08 |  |
| LDL-cholesterol, mmol/l | 2.2 (1.8-2.8) | | 2.2 (1.8-2.7) | | 2.5 (1.9-3.1) | 0.23 |  |
| HDL-cholesterol, mmol/l | 1.2 (1.0-1.5) | | 1.2 (1.0-1.5) | | 1.4 (1.2-1.7) | 0.15 |  |
| hsCRP, mg/l | 1.8 (0.9-3.4) | | 1.5 (0.8-3.1) | | 3.2 (1.9-4.9) | 0.01 |  |
| **Coagulation parameters** |  |  |  |  |  |  |  |
| INR | 0.99 (0.95-1.04) | | 0.98 (0.95-1.05) | | 0.99 (0.97-1.03) | 0.81 |  |
| APTT, s | 27.4 (25.7-29.3) | | 27.6 (26.2-29.7) | | 26.6 (25.4-29.1) | 0.38 |  |
| Fibrinogen, g/l | 3.0 (0.6) | | 2.9 (0.6) | | 3.1 (0.6) | 0.27 |  |
| Peak thrombin, nM | 227.2 (45.1) | | 224.6 (46.2) | | 237.3 (40.6) | 0.29 |  |
| Plasminogen activity, % | 107 (99 - 120) | | 108 (99.5 - 122.5) | | 102.5 (92 - 114) | 0.27 |  |
| tPA antigen, ng/ml | 11.6 (2.9) | | 11.6 (2.8) | | 11.5 (3.4) | 0.91 |  |
| PAI-1 antigen, ng/ml | 31.4 (28.6-37.9) | | 30.8 (28.4-37.5) | | 32.3 (28.9-38.9) | 0.69 |  |
| α2-antiplasmin, % | 109 (100 - 119) | | 109 (100 - 121) | | 109 (99 - 116) | 0.49 |  |
| TAFI activity, % | 96 (84 - 106) | | 90 (83-101) | | 104.5 (98-110) | <0.001 |  |
| Thrombomodulin antigen, ng/ml | 2.9 (2.4 - 3.5) | | 2.9 (2.4 - 3.3) | | 2.7 (2.4 - 3.6) | 0.98 |  |
| FXI, % | 107.8 (12.4) | | 103.2(9.1) | | 125.2 (5.2) | <0.001 |  |
| FXI>120% | 18 (20.9) | |  | |  |  |  |
| **Fibrin clot properties** |  |  |  |  |  |  |  |
| lag phase, s | 42.7 (4.6) | | 42.5 (4.7) | | 43.2 (4.4) | 0.58 |  |
| ΔAbs max, 405 nm | 0.81 (0.77-0.86) | | 0.81 (0.77-0.86) | | 0.82 (0.80-0.84) | 0.35 |  |
| Ks, x10^-9^cm^2^ | 7.1 (0.8) | | 7.1 (0.8) | | 6.9 (0.7) | 0.30 |  |
| Compaction, % | 44.5 (6.1) | | 44.8 (6.4) | | 43.4 (5.1) | 0.42 |  |
| CLT, min | 93.0 (18.1) | | 92.8 (19.0) | | 93.6 (14.6) | 0.87 |  |
| t50_%_, min | 9.9 (9.0-10.7) | | 9.9 (8.9-10.5) | | 10 (9.5-10.8) | 0.19 |  |
| D-D_max_, mg/l | 3.88 (3.62-4.18) | | 3.87 (3.60-4.11) | | 3.89 (3.74-4.31) | 0.20 |  |
| D-D_rate_, mg/l/min | 0.071 (0.005) | | 0.071 (0.005) | | 0.069 (0.005) | 0.04 |  |

Abbreviations: ΔAbs – maximum absorbance at plateau; APTT - Activated Partial Thromboplastin Time; CLT – clot lysis time; D-D_max_ – maximum D-dimer concentration; D-D_rate_ – maximum rate of increased in D-dimer concentration; HbA1C – glycated hemoglobin; HDL – high-density lipoprotein; hsCRP – high sensitivity C-reactive protein; INR – international normalized ratio; Ks – permeability coefficient; LDL– low-density lipoprotein; PAI-1 – Plasminogen activator inhibitor – 1; t_50_ - time required for a 50 decrease in clot turbidity; TAFI – thrombin-activatable fibrinolysis inhibitor; tPA – tissue plasminogen activator;

**Table S2 Patient characteristics with regard to clinical endpoints in patients with type 2 diabetes mellitus and coronary artery disease**

|  |  | MI+stroke+CV death | |  | CV death |  |
| --- | --- | --- | --- | --- | --- | --- |
|  | No, n=71 (82.6%) | Yes, n=15 (17.4%) | *p* value | No, n=73 (84.9%) | Yes, n=13 (15.1%) | *p* value |
| Age, years, n (%) | 66.7 (7.9) | 70.1 (8.6) | 0.15 | 66.8 (7.8) | 70.2 (9.1) | 0.17 |
| Female, n (%) | 26 (36.6) | 5 (33.3) | 0.81 | 26 (35.6) | 5 (38.5) | 1.00 |
| Body mass index, kg/m2 | 31.5 (5.0) | 30.0 (5.0) | 0.30 | 31.5 (4.9) | 29.9 (5.4) | 0.30 |
| Current smoker, n (%) | 5 (7.0) | 2 (13.3) | 0.60 | 5 (6.8) | 2 (15.4) | 0.29 |
| Time since diabetes diagnosis, years | 6 (3-10) | 8 (1-11) | 0.81 | 6 (3-10) | 8 (1-10) | 0.80 |
| **Comorbidities, n (%)** |  |  |  |  |  |  |
| Hypertension | 67 (94.4) | 12 (80.0) | 0.10 | 68 (93.2) | 11 (84.6) | 0.29 |
| Prior myocardial infarction | 19 (26.8) | 6 (40.0) | 0.35 | 19 (26.0) | 6 (46.2) | 0.19 |
| Prior stroke | 1 (1.4) | 1 (6.7) | - | 1 (1.4) | 1 (7.7) | - |
| Retinopathy | 14 (19.7) | 4 (26.7) | 0.51 | 15 (20.5) | 3 (23.1) | 1.00 |
| Nephropathy | 10 (14.1) | 6 (40) | 0.03 | 10 (13.7) | 6 (46.2) | 0.01 |
| Neuropathy | 15 (21.1) | 4 (26.7) | 0.73 | 15 (20.5) | 4 (30.8) | 0.47 |
| **Pharmacotherapy, n (%)** |  |  |  |  |  |  |
| Acetylsalicylic acid | 66 (93.0) | 13 (86.7) | 0.60 | 68 (93.2) | 11 (84.6) | 0.29 |
| Clopidogrel | 9 (12.7) | 1 (6.7) | 1.00 | 9 (12.3) | 1 (7.7) | 1.00 |
| Statin | 63 (88.7) | 13 (86.7) | 1.00 | 65 (89) | 11 (84.6) | 0.64 |
| Sulfonylurea | 30 (42.3) | 10 (66.7) | 0.09 | 32 (43.8) | 8 (61.5) | 0.37 |
| Metformin | 46 (64.8) | 4 (26.7) | 0.01 | 46 (63) | 4 (30.8) | 0.03 |
| Insulin | 18 (25.4) | 4 (26.7) | 1.00 | 18 (24.7) | 4 (30.8) | 0.73 |
| **Basic laboratory data** |  |  |  |  |  |  |
| White blood count, x 10^9^/l | 7.0 (1.3) | 8.4 (1.8) | <0.001 | 7.0 (1.3) | 8.5 (1.9) | <0.001 |
| Neutrophiles, x 10^9^/l | 60.2 (8.5) | 66.2 (11.6) | 0.03 | 60.2 (8.4) | 67.2 (12.1) | 0.01 |
| Lymphocytes, x 10^9^/l | 27.8 (7.5) | 22.9 (10.0) | 0.04 | 27.8 (7.5) | 21.8 (10.1) | 0.01 |
| Platelets, x 10^9^/l | 207 (183-252) | 178 (152-254) | 0.22 | 206 (183-252) | 178 (152-254) | 0.34 |
| Hemoglobin, g/dl | 13.8 (1.1) | 13.7 (1.5) | 0.76 | 13.9 (1.1) | 13.4 (1.4) | 0.25 |
| GFR, ml/min | 79.5 (20.2) | 66.7 (23.6) | 0.03 | 79.5 (19.9) | 64.9 (24.9) | 0.02 |
| Fasting glucose, mmol/l | 5.7 (4.9-7.4) | 5 (4.1-6.2) | 0.08 | 5.7 (4.9-7.3) | 5 (4.1-6.2) | 0.09 |
| HbA1c, % | 6.5 (6.1-7.3) | 6.6 (6.2-8.3) | 0.69 | 6.5 (6.1-7.3) | 6.6 (6.2-8.3) | 0.60 |
| Total cholesterol, mmol/l | 4.3 (3.5-4.8) | 3.5 (3.2 - 3.8) | 0.03 | 4.3 (3.5-4.8) | 3.4 (3.2 - 3.6) | 0.02 |
| LDL-cholesterol, mmol/l | 2.3 (1.9-2.9) | 1.7 (1.4-2.1) | 0.01 | 2.3 (1.9-2.8) | 1.7 (1.4-1.9) | <0.001 |
| HDL-cholesterol, mmol/l | 1.3 (1.1-1.5) | 1.2 (0.8-1.4) | 0.14 | 1.3 (1.1-1.5) | 1.2 (1.0-1.4) | 0.27 |
| hsCRP, mg/l | 1.5 (0.8-2.9) | 3.5 (3.1-6.3) | <0.001 | 1.5 (0.9-3.0) | 4.2 (3.1-6.3) | <0.001 |
| **Coagulation parameters** |  |  |  |  |  |  |
| INR | 0.98 (0.95-1.03) | 0.99 (0.96-1.07) | 0.78 | 0.98 (0.95-1.03) | 0.99 (0.96-1.06) | 0.92 |
| APTT, s | 27.9 (26.0-30.0) | 26.1 (25.2-27.1) | 0.04 | 27.9 (25.9-29.8) | 26.5 (25.2-27.1) | 0.10 |
| Fibrinogen, g/l | 2.9 (0.6) | 3.2 (0.6) | 0.09 | 2.9 (0.6) | 3.2 (0.6) | 0.10 |
| Peak thrombin, nM | 224.3 (44.1) | 241.1 (49.0) | 0.19 | 225.2 (45.0) | 238.6 (45.9) | 0.33 |
| Plasminogen activity, % | 107 (100-120) | 105 (85-126) | 0.49 | 107 (100-120) | 104 (85-110) | 0.25 |
| tPA antigen, ng/ml | 11.4 (2.6) | 12.3 (4.2) | 0.29 | 11.4 (2.6) | 12.5 (4.3) | 0.24 |
| PAI-1 antigen, ng/ml | 30.8 (28.0-37.5) | 31.9 (29.0-39.2) | 0.60 | 30.8 (28.1-37.5) | 31.9 (29.0-39.0) | 0.79 |
| α2-antiplasmin, % | 108 (100 - 117) | 116 (100 - 121) | 0.43 | 108 (100 - 117) | 116 (102 - 121) | 0.38 |
| TAFI activity, % | 94 (85-106) | 97 (83-109) | 0.96 | 94 (85-106) | 97 (83-103) | 0.98 |
| Thrombomodulin antigen, ng/ml | 2.9 (2.4-3.3) | 2.9 (2.4-3.7) | 0.90 | 2.9 (2.4- 3.3) | 2.9 (2.4-3.7) | 0.82 |
| FXI, % | 105.5 (10.8) | 118.6 (13.8) | <0.001 | 106.1 (11.2) | 117.5 (14.5) | <0.001 |
| FXI>120% | 9 (12.7) | 9 (60.0) | <0.001 | 11 (15.1) | 7 (53.8) | 0.01 |
| **Fibrin clot properties** |  |  |  |  |  |  |
| lag phase, s | 42.9 (4.7) | 41.6 (4.3) | 0.32 | 42.8 (4.7) | 42.0 (4.1) | 0.56 |
| ΔAbs max, 405 nm | 0.80 (0.77-0.86) | 0.82 (0.79-0.87) | 0.30 | 0.80 (0.77-0.85) | 0.83 (0.81-0.87) | 0.14 |
| Ks, x10^-9^cm^2^ | 7.1 (0.8) | 6.7 (0.8) | 0.049 | 7.1 (0.8) | 6.7 (0.8) | 0.10 |
| Compaction, % | 44.7 (6.3) | 43.2 (5.4) | 0.37 | 44.7 (6.2) | 43.1 (5.8) | 0.37 |
| CLT, min | 92.6 (17.4) | 94.9 (21.8) | 0.66 | 92.6 (17.5) | 94.9 (22.2) | 0.68 |
| t50_%_, min | 9.8 (8.9 - 10.6) | 10.2 (9.8 - 10.9) | 0.049 | 9.9 (9.0-10.4) | 10.2 (9.8-10.9) | 0.06 |
| D-D_max_, mg/l | 3.78 (3.60-4.07) | 4.26 (3.89-4.64) | <0.001 | 3.8 (3.6-4.1) | 4.3 (3.9-4.6) | <0.001 |
| D-D_rate_, mg/l/min | 0.072 (0.005) | 0.067 (0.005) | <0.001 | 0.072 (0.005) | 0.067 (0.005) | <0.001 |

Abbreviations: see Table S1

**Table S3 Multivariable analysis – clinical endpoints in patients with co-existing coronary artery disease**

| MI+stroke+CV mortality | | | |  |  |  | CV mortality |  |
| --- | --- | --- | --- | --- | --- | --- | --- | --- |
|  | HR | 95% CI | *p* value |  |  | HR | 95% CI | *p* value |
| Age | 1.10 | (1.01-1.19) | 0.02 |  | Age | 1.09 | (1.00-1.19) | 0.046 |
| Female | 0.41 | (0.10-1.70) | 0.22 |  | Female | 0.56 | (0.12-2.54) | 0.45 |
| LDL-C | 0.42 | (0.18-0.98) | 0.04 |  | LDL-C | 0.33 | (0.12-0.85) | 0.02 |
| D-D_max_ | 13.72 | (3.08-61.07) | 0.001 |  | D-D_max_ | 18.98 | (3.63-99.17) | <0.001 |
| FXI>120% | 10.24 | (3.25-32.26) | <0.001 |  | FXI>120% | 9.23 | (2.67-31.95) | <0.001 |

Abbreviations: see Table S1

**Table S4 Baseline patient characteristics with regard to FXI levels and the composite endpoint in patients with type 2 diabetes mellitus, without coronary artery disease**

|  |  |  |  |  | MI+stroke+CV death | |  |
| --- | --- | --- | --- | --- | --- | --- | --- |
|  | All patients without CAD, n=47 | FXI ≤ 120,  n=40 (85.1%) | FXI >120,  n=7 (14.9%) | *p* value | No,  n=41 (87.2%) | Yes,  n=6 (12.8%) | *p* value |
| Age, years, n (%) | 63.7 (7.4) | 63.0 (7.3) | 67.6 (7.3) | 0.13 | 63.1 (7.1) | 67.8 (8.3) | 0.14 |
| Female, n (%) | 23 (48.9%) | 18 (45.0) | 5 (71.4) | 0.25 | 21 (51.2) | 2 (33.3) | 0.66 |
| Body mass index, kg/m2 | 33.2 (5.4) | 33.7 (5.5) | 30.2 (3.9) | 0.12 | 33.6 (5.5) | 30.5 (3.9) | 0.19 |
| Current smoker, n (%) | 5 (10.6) | 5 (12.5) | 0 | 1.00 | 5 (12.2) | 0 (0) | 1.00 |
| Time since diabetes diagnosis, years | 4 (2-11) | 4 (2-11.5) | 3 (2-5) | 0.95 | 3 (2-9) | 4.5 (3-21) | 0.19 |
| **Comorbidities, n (%)** |  |  |  |  |  |  |  |
| Hypertension | 47 (100) | - | - | - | - | - | - |
| Retinopathy | 3 (6.4) | 3 (7.5) | 0 (0) | 1.00 | 2 (4.9) | 1 (16.7) | 0.34 |
| Nephropathy | 7 (14.9) | 6 (15.0) | 1 (14.3) | 1.00 | 6 (14.6) | 1 (16.7) | 1.00 |
| Neuropathy | 5(10.6) | 5 (12.5) | 0 (0) | 1.00 | 5 (12.2) | 0 (0) | 1.00 |
| **Pharmacotherapy, n (%)** |  |  |  |  |  |  |  |
| Acetylsalicylic acid | 26 (55.3) | 24 (60.0) | 2 (28.6) | 0.22 | 24 (58.5) | 2 (33.3) | 0.39 |
| Statin | 30 (63.8) | 24 (60.0) | 6 (85.7) | 0.40 | 25 (61.0) | 5 (83.3) | 0.40 |
| Sulfonylurea | 16 (34.0) | 13 (32.5) | 3 (42.9) | 0.68 | 13 (31.7) | 3 (50.0) | 0.40 |
| Metformin | 30 (63.9) | 25 (62.5) | 5 (71.4) | 1.00 | 27 (65.9) | 3 (50.0) | 0.65 |
| Insulin | 11 (23.4) | 10 (25.0) | 1 (14.3) | 1.00 | 9 (22.0) | 2 (33.3) | 0.61 |
| **Basic laboratory data** |  |  |  |  |  |  |  |
| White blood count, x 10^9^/l | 6.9 (1.3) | 6.9 (1.4) | 6.6 (0.9) | 0.59 | 6.9 (1.4) | 6.4 (1.1) | 0.34 |
| Neutrophiles, x 10^9^/l | 60.0 (7.3) | 59.4 (7.6) | 63.2 (5.4) | 0.22 | 59.5 (7.6) | 63.6 (4.5) | 0.20 |
| Lymphocytes, x 10^9^/l | 28.7 (6.8) | 29.1 (7.2) | 26.7 (3.7) | 0.40 | 29.3 (7.0) | 24.7 (3.0) | 0.12 |
| Platelets, x 10^9^/l | 210 (171-266) | 207 (165-255) | 248 (209 - 343) | 0.07 | 210 (171-258) | 225 (193-269) | 0.53 |
| Hemoglobin, g/dl | 13.8 (1.2) | 13.8 (1.2) | 13.4 (1.3) | 0.45 | 13.8 (1.1) | 13.8 (1.7) | 0.98 |
| GFR, ml/min | 80.5 (19.5) | 79.8 (19.8) | 85.0 (18.0) | 0.51 | 79.1 (19.0) | 90.6 (21.5) | 0.18 |
| Fasting glucose, mmol/l | 6.4 (5.8-7.4) | 6.6 (5.8-7.5) | 6.3 (5.8-7.3) | 0.87 | 6.7 (5.9-7.4) | 6.1 (5.3-6.5) | 0.35 |
| HbA1c, % | 6.2 (6.0-7.1) | 6.3 (6.0-7.1) | 6.2 (6.1-6.7) | 0.60 | 6.2 (6.0-6.9) | 6.2 (6.1-7.1) | 1.00 |
| Total cholesterol, mmol/l | 4.4 (3.7-5.5) | 4.2 (3.6-5.3) | 5.8 (5.0-6.3) | 0.01 | 4.3 (3.7-5.5) | 5.0 (4.6-5.1) | 0.64 |
| LDL-cholesterol, mmol/l | 2.6 (2.0-3.4) | 2.4 (1.9-3.2) | 3.6 (3.0-4.4) | <0.001 | 2.5 (2.0-3.4) | 2.9 (2.8-3.2) | 0.38 |
| HDL-cholesterol, mmol/l | 1.4 (1.2-1.7) | 1.4 (1.2-1.7) | 1.6 (1.3-1.7) | 0.52 | 1.4 (1.2-1.7) | 1.5 (1.3-1.7) | 0.89 |
| hsCRP, mg/l | 2.0 (1.2-5.0) | 2.0 (1.3-4.6) | 2.0 (0.8-5.7) | 0.81 | 2.3 (1.3-5.2) | 1.6 (0.8-2.0) | 0.07 |
| **Coagulation parameters** |  |  |  |  |  |  |  |
| INR | 0.99 (0.93-1.02) | 0.99 (0.93-1.02) | 0.98 (0.94-1.00) | 0.84 | 0.97 (0.93-1.01) | 1.00 (0.98-1.02) | 0.30 |
| APTT, s | 27.1 (26.2-28.8) | 27.1 (25.7-28.6) | 27.8 (26.5-29.8) | 0.32 | 27.1 (26.2-28.7) | 27.8 (26.3-29.8) | 0.68 |
| Fibrinogen, g/l | 3.3 (0.6) | 3.2 (0.6) | 3.5 (0.4) | 0.19 | 3.3 (0.6) | 3.4 (0.5) | 0.72 |
| Peak thrombin, nM | 249.4 (43.7) | 248.6 (43.7) | 254.2 (46.4) | 0.75 | 246.1 (42.1) | 272.0 (51.5) | 0.18 |
| Plasminogen activity, % | 104 (97-113) | 105 (97-113) | 101 (97-121) | 0.76 | 105 (97-113) | 100 (98-113) | 0.84 |
| tPA antigen, ng/ml | 11.5 (2.6) | 11.6(2.6) | 10.7 (2.8) | 0.39 | 11.6 (2.7) | 10.3 (2.0) | 0.25 |
| PAI-1 antigen, ng/ml | 33.6 (29.0-39.1) | 33.6 (28.9-39.4) | 34.0 (30.0-36.3) | 0.82 | 33.6 (28.7-39.3) | 34.6 (33.6-36.3) | 0.69 |
| α2-antiplasmin, % | 102 (94-110) | 102 (93-111) | 102 (97-110) | 0.85 | 101 (95-109) | 106 (94-113) | 0.67 |
| TAFI activity, % | 103 (96-113) | 103 (96-112) | 110 (97-139) | 0.38 | 103 (96-112) | 106 (97-116) | 0.71 |
| Thrombomodulin antigen, ng/ml | 3.0 (2.7-3.5) | 3.0 (2.8-3.5) | 2.8 (2.1-4.1) | 0.53 | 3.0 (2.8-3.9) | 2.5 (2.1-3.3) | 0.13 |
| FXI, % | 106.5 (14.3) |  |  |  | 104.0 (10.7) | 123.8 (23.8) | <0.001 |
| FXI>120% | 7 (14.9) |  |  |  | 3 (7.3) | 4 (66.7) | <0.001 |
| **Fibrin clot properties** |  |  |  |  |  |  |  |
| lag phase, s | 43.8 (4.2) | 43.9 (4.3) | 43.1 (4.3) | 0.68 | 43.9 (4.3) | 42.8 (4.3) | 0.57 |
| ΔAbs max, 405 nm | 0.82 (0.80-0.85) | 0.82 (0.80-0.86) | 0.81 (0.78-0.85) | 0.75 | 0.81 (0.80-0.85) | 0.85 (0.80-0.85) | 0.43 |
| Ks, x10^-9^cm^2^ | 7.3 (0.9) | 7.3 (0.9) | 7.5 (0.8) | 0.60 | 7.3 (0.9) | 7.2 (1.0) | 0.85 |
| Compaction, % | 44.5 (5.6) | 45.1 (5.5) | 41.6 (5.5) | 0.13 | 45.0 (5.5) | 41.3 (5.5) | 0.13 |
| CLT, min | 96.1(16.2) | 96.0 (16.5) | 96.6 (15.6) | 0.94 | 96.6 (16.2) | 92.5 (17.6) | 0.57 |
| t50_%_, min | 9.7 (8.8-10.2) | 9.8 (8.8-10.4) | 9.2 (9.0-10.1) | 0.55 | 9.7 (8.8-10.1) | 10.0 (9.4-10.5) | 0.40 |
| D-D_max_, mg/l | 3.85 (3.63-4.09) | 3.85 (3.62-4.03) | 3.73 (3.69-4.12) | 0.99 | 3.85 (3.65-4.04) | 3.70 (3.63-4.09) | 0.57 |
| D-D_rate_, mg/l/min | 0.069 (0.004) | 0.069 (0.004) | 0.071 (0.004) | 0.17 | 0.069 (0.004) | 0.071 (0.003) | 0.45 |

Abbreviations: see Table S1
